# Supplementary material for: Are carbon emissions trading and green financial instruments synergistic? -Comprehensive quantitative research based on content analysis
Source: PLoS One. 2024 Mar 7;19(3):e0298601. doi: 10.1371/journal.pone.0298601 (PMC10919601; doi:10.1371/journal.pone.0298601)
Supplement: S1 Data — (ZIP) [file pone.0298601.s001.zip › Data/地级市Z_F工作报告.zip!/╜¡╬≈/╥╦┤║╩╨/└┤╫╘╣1⁄2╓┌║┼í╛┬φ┐╦╩2╛▌═°í┐_2007.docx]

各位代表：

　　现在，我代表市人民政府，向大会作政府工作报告，请予审议，并请各位市政协委员和列席会议的同志提出意见。

　　一、2006年工作简要回顾

　　过去的一年，我们在省委、省政府和市委的正确领导下，以邓小平理论和“三个代表”重要思想为指导，深入贯彻党的十六大和十六届五中、六中全会精神，立足科学发展，加快改革创新，推动和谐进步，全面完成了市一届人大九次会议确定的各项工作目标，实现了“十一五”的良好开局。

　　经济保持平稳快速发展，运行质量跃上新水平。完成生产总值439.72亿元，比上年增长13.2%，人均生产总值突破1000美元，达到1042美元。财政总收入38.68亿元，增长25.1%，增幅为十年来最高；其中地方财政收入21.83亿元，增长20.2%，增幅居全省第四。全社会固定资产投资202.52亿元，增长21.4%。社会消费品零售总额131.48亿元，增长14.4%。县域经济快速发展，实现了财政收入县县过亿元、丰城超10亿元的历史性突破。丰城、樟树、奉新、上高、高安受到省委、省政府的表彰，占全省受表彰县市区总数的七分之一。

　　工业化和城市化进程加快，新农村建设迈出新步伐。规模以上工业完成增加值103.99亿元，增长34.9%；实现销售收入300.9亿元，增长52.5%；实现利税34.57亿元，增长72.6%；五大支柱产业完成增加值占全部工业的67.9%。9个工业园区均通过国家审核和省政府审批，升格为省级工业园，入园企业达到993户，实现税收14.54亿元，增长66.9%。丰城、奉新、上高工业园获全省先进工业园区称号。城镇建设步伐加快，城市化率达34.5%。宜春中心城被评为“全国绿化模范城市”、“国家园林城”、“全国道路交通一等管理水平城市”和首届“江西省文明城市”，城市管理工作连续四年居全省第一。以“和谐小康家园行动”为主题的社会主义新农村建设顺利展开，570个自然村试点工作成效明显。发放粮食直补、农业生产资料补贴、良种补贴2.47亿元，粮食总产、水稻总产和单产均创历史新高。农业产业化快速推进，拥有市级以上龙头企业94家，十大主导产业实现综合产值60.52亿元、税收2.44亿元，分别增长32.8%、59.5%。林业产业发展迅速，营造高产油茶林4.31万亩，改造毛竹林22.66万亩，栽植杨树2200余万株。基础设施建设不断加强，完成病险水库除险加固332座，修复水毁工程587处，新建小型水源工程280处、供水工程74处，受益人口5万余人，由国家投资的7个土地整理项目通过国土资源部评审立项；沼气工程顺利推进，农村户用沼气池保有量4.5万个，规模养殖场环保沼气工程达到126处；武吉高速公路宜春境内段征地拆迁基本完成，宜安公路正式动工，整修和改善市养公路171公里，完成11条线路的安保工程建设，县乡水泥(油)路竣工里程1353.7公里，新增通水泥(油)路建制村284个。

　　各项改革深入推进，开放型经济和民营经济迸发新活力。在市本级试点基础上，部门预算改革向县级延伸，国库集中收付制度改革全面铺开，会计集中代理核算更趋规范，“乡财县代管”试点达到71个乡镇。林业产权制度改革基本完成，水利工程管理体制改革稳步推进，国有粮食购销企业改革正式启动。市直事业单位改革进展顺利，精简机构92个，精简率36.4%。大力优化发展环境，积极组织和参加一系列招商引资和对外经贸活动，引进重大工业项目44项，实际利用外资2.36亿美元，增长11.1%；引进市外5000万元以上工业项目资金95.76亿元；外贸出口1.5亿美元，增长36.1%，我市和上高荣获全省开放型经济发展综合奖。以民营经济为主体的经济增长格局已经形成，民营企业注册资金119.7亿元，纳税19.5亿元，占据财政收入的“半壁江山”。

　　经济社会统筹发展，各项社会事业有了新进步。科技创新氛围更加浓厚，知识产权保护成效明显，专利申请量列全省设区市之首。中心城大文化产业发展布局加紧实施，宜春晚报更名为赣西晚报，覆盖面扩大至新余、萍乡；袁州谯楼、樊城堆、鸣水桥、朱轼墓、湘鄂赣革命根据地旧址被核定为全国重点文物保护单位。1632所农村中小学远程教育设施全部安装完成，高安被确定为农村远程教育项目国家级专家培训点，上高在省政府教育督导评估中得分第一，全市第一个博士后科研工作站落户宜春学院。中心城区国家卫生城创建全面推进。县市区疾控中心建设基本完成，新型农村合作医疗试点工作进展顺利，在全省率先开展新农村卫生所建设。成功承办了世界杯藤球赛、全国摩托艇精英赛暨艺术滑水表演赛等17项国际国内赛事；在第十二届省运会上，金牌、奖牌总数和团体总分均居全省第二，取得历史性突破。成功承办2006中国光彩事业宜春行暨明月山森林温泉旅游节，旅游业发展加速，实现总收入22.65亿元，接待国内游客383.8万人次，增长17.8%；接待海外游客突破1万人次，增长29.6%，外汇收入354万美元，增长37.6%。

　　城乡居民生活不断改善，和谐稳定取得新成绩。城镇居民人均可支配收入9153元，增加923元；农民人均纯收入3653元，增加223元。城乡居民人均储蓄存款6152元，增加663元；城乡居民人均住房面积分别达到28.13平方米和36.71平方米。新增城镇从业人员8.53万人，下岗失业人员再就业3.63万人，“4050”群体再就业6487人，城镇登记失业率控制在3.9%。新增小额担保贷款基金2060万元，担保贷款1.25亿元、额度超过前三年的总和。免费职业培训4.9万人，创业培训2745人；免费职业介绍12.4万人，跨省劳务输出82.3万人。养老、医疗、失业、工伤、生育保险参保人数分别达到26.9万人、29.2万人、26.1万人、18.4万人、11万人，五项基金总收入7.72亿元，增长26.8%。人口和计生工作进一步加强，人口自然增长率控制在7.34‰。环境保护成效明显，7条主要河流地表水环境质量稳定在III类水标准以上，中心城区环境空气质量达到一级优的天数明显增加。认真落实安全生产责任制，深化各项专项整治，安全生产态势平稳。积极畅通信访渠道，完善工作机制，大量矛盾纠纷和不稳定因素消除在萌芽状态。深入开展 “扫黑除恶”、侦破命案、打击“两抢一盗”等活动，和谐平安创建扎实推进，群众的公共安全感进一步提升。

　　民主法制建设继续推进，依法行政水平不断提高。坚持向人大报告政府工作，加强与政协联系，认真接受人大及其常委会的法律监督和政协的民主监督，181件人大代表的建议、政协委员的提案全部办复。“五五”普法全面启动，法律援助和法律服务效能继续提升。公务员登记工作进展顺利，廉政建设进一步加强，行政监察、效能监察和审计监督成效明显。国防力量和民兵预备役建设、人民防空、国家安全、民族宗教、广播电视、外事侨务、涉台事务、气象服务、防震减灾、国有资产管理、妇女儿童、统计、档案、扶贫、扶残、老龄和红十字会等各项工作都取得新的进步。

　　各位代表！过去一年成就的取得，是省委、省政府和市委正确领导的结果，是全市人民团结奋斗、积极进取的结果，是与历届政府打下的扎实基础分不开的。在此，我谨代表市人民政府，向所有关心、支持和参与宜春发展的广大工人、农民、知识分子、驻宜部队、武警官兵、公安干警、中央和省驻宜单位、各民主党派、工商联、无党派人士、各人民团体、离退休老同志和海内外朋友，表示衷心的感谢和崇高的敬意！

在看到成绩的同时，我们也清醒地认识到一些深层次的矛盾和问题，主要是：经济社会发展的总体水平不高，竞争力不强，资源约束和环境压力增大；市本级财力和开发区实力不强，中心城规模不大，辐射和带动力偏弱；群众生活水平偏低，农民增收难度较大，困难群众生产生活还有不少问题亟待解决；政务环境和行政效能还需进一步优化，等等。对这些问题，我们将高度重视，在改革发展中努力克服和解决。

　　二、2007年工作目标与任务

　　2007年是全面贯彻省第十二次党代会、市第二次党代会和市二届人大一次会议精神的第一年，也是深入落实科学发展观、推进和谐宜春建设的重要一年。做好今年各项工作，必须正确判断当前面临的发展形势，在宏观调控中抢抓机遇，在激烈竞争中奋勇争先，更加注重提升发展的质量与速度，更加注重增强发展的动力与后劲，更加注重优化发展的环境与效能。

　　今年政府工作的总体要求是：以邓小平理论和“三个代表”重要思想为指导，以科学发展观统领全局，紧紧围绕“在全省整体实力前移、局部工作站前列”的总体目标，坚持民生为本、开放为先、创新为要、和谐为重、行政为民，深入实施“心圈廊”发展战略，突出决战工业园、建设新农村、做大市本级，加快改革创新，倾力改善民生，促进和谐平安，推动全市经济社会又好又快地发展。经济社会发展总体目标为：生产总值增长13%，财政总收入和地方财政收入分别增长20%，规模以上工业增加值增长25%，全社会固定资产投资增长25%，实际利用境外资金增长20%，引进市外工业资金增长25 %，外贸出口增长26%，社会消费品零售总额增长14%，农民人均纯收入增长7.2%、达到3915元以上，城镇居民人均可支配收入增长8%、增加732元，城镇登记失业率控制在4.5%以内，人口自然增长率控制在8.01‰以内，城镇化率达到36%。 为实现上述目标，今年要重点抓好以下六方面的工作：

　　第一，大力提升区域经济发展层次。按照“丰富内涵、协作联动、三位一体”的要求，加速推进“心圈廊”格局的建设。以“三大战役”为发展契机，做大中心城区规模，加快人气、商气和财气的聚集；壮大机械电子、新型材料、医药和油茶加工产业；打响明月山温泉风景名胜区“奇山、妙泉、名禅、神农”旅游品牌，全面促进“心”发展。以丰城的煤、电、禽，樟树的药、酒、盐，高安的机械、建材、货运产业为发展主体，继续壮大“圈”实力。以上高的食品、鞋革加工，奉新的纺织服装，靖安的旅游、有色金属加工，万载、铜鼓、宜丰的山区生态有机产品为发展重点，打造320国道和山区特色经济两“廊”特色。以市场为导向，以利益联结为纽带，以产业整合为重点，积极探索“心”、“圈”、“廊”之间优势互补、协作共进的发展机制，逐步建成特色鲜明的区域经济体系。强化“心圈廊”发展内涵，积极构筑特色经济板块，加快市本级医药、樟树盐化工、丰城电能气、高安建筑陶瓷、奉新纺织服装、上高鞋业六大基地建设，推进医药、食品、建材、机械、电力能源五大支柱产业发展，重点抓好一批龙头骨干企业新扩改项目建设，努力培植100户销售收入上亿元企业，力争五大产业销售收入和利税均增长30%以上，规模以上工业综合效益指数达到165%以上，新增省级名牌产品和著名商标各10个，中国名牌产品和中国驰名商标各1个。

　　第二，合力唱响全民创业主旋律。进一步放宽准入领域、降低注册门槛、简化开办手续，开展职业技能培训，鼓励支持公职人员、军队转业干部、退役士兵、下岗失业人员、大中专毕业生、农民等各类人员自主创业，在市内兴办各类经济实体，鼓励宜春籍在外创业或务工人员返乡创业。依托工业园区或现有闲置土地、厂房，开辟小企业创业基地，积极发展专业市场、专业街、特色产业基地等各种创业平台，完善社会化服务体系，为创业者提供优质服务。全面落实各项扶持政策，支持企业加大投入，支持发展农业产业化龙头企业，支持创办第三产业，支持发展高新技术产业，支持参与国有经济调整和国有企业重组，支持创办资源综合利用型企业，支持小企业发展。进一步优化发展环境，扎实开展“作风建设推进年”活动，严肃查处损害发展环境的行为，以行政服务水平和政府公信力的提升，促进全民创业和民营经济健康发展。全市民营经济实现企业增加值、营业收入分别增长20%，实现税收增长25%。

　　第三，着力增强经济发展基础和后劲。以“植产业之树、造企业之林”为定位，狠抓产业招商，大力突破外资增量，集中优势力量和政策资源招“大”引“强”，确保全年引进重大项目40个；优化外贸出口结构，提高出口质量，完善服务体系，努力扩大出口创汇；积极拓展外经市场，稳步推进对外经济合作。精心组织项目策划，密切与上级有关部门的沟通联系，争取更多政策、项目和资金支持。健全信用担保体系，大胆创新激励机制，推进银企合作，扩大工业和民营经济信贷资金投放；加强异地融资，积极争取外地金融机构落户；加快企业诚信体系建设，规范企业信用征集、发布、查询和评级管理，建设中小企业信息化平台。健全城乡基础设施，支持配合武吉高速公路和宜安公路建设，实施320国道万载至大城段准高速公路项目，加快高安至胡家坊一级公路和丰城剑邑大桥建设，做好明月山机场、105国道丰城至樟树一级公路等项目的前期工作；认真抓好丰电公司二期和丰龙煤井工程、奉新和丰城两个220千伏变电站项目建设，加快洪屏抽水蓄能电站前期工作；继续抓好筠安堤、三湖联圩、赣西肖江堤、药湖大联圩等在建工程建设，推进锦北、潦河、丰东、袁北四大灌区续建配套和节水改造，基本完成列入省病险水库规划的小（一）型、小（二）型水库的除险加固。

　　第四，强力推进社会主义新农村建设。以发展农村生产力、增加农民收入为中心，加快现代农业建设。继续落实惠农政策，加强农业综合开发，实施“造地增粮富民工程”，稳定粮食生产。加强农业新技术、新品种的推广应用，大力发展具有区域特色的农业机械化，继续抓好10个水稻全程机械化示范村建设。加强无公害农产品、绿色食品、有机农产品的产地认定、申报认证和农产品质量安全检测工作。高度重视重大动物疫病防控，杜绝重大动物疫病的发生和蔓延。继续推进农业产业化经营，着力培育和扶持一批起点高、规模大、带动面广、竞争力强的龙头企业，促进主导产业、龙头企业、生产基地和服务体系健康发展，确保十大主导产业综合产值和税收均增长20%以上。大力发展农民合作经济组织，提高农业生产的组织化程度。积极实施长防林、退耕还林、生态公益林等林业工程，大力发展以杨树为主的速生丰产林、高产油茶林、毛竹丰产林。以“和谐小康家园行动”为总抓手，以“四兴一强”为目标，以试点村为重点，继续推进新农村建设村庄整治工作。科学编制村庄规划，全面完成平原200人以上、山区100人以上的村庄规划；按照“三清、三改、三配套”的要求，大力实施“乡村清洁工程”和沼气富民工程，重点解决“走平坦路、喝干净水、上卫生厕、住整洁房屋、用洁净能源”问题；继续实施农村饮水安全工程，解决15万人用水安全问题；进一步完善村级党组织领导的充满活力的村民自治机制，加强农村村落社区建设，健全农村民主选举、民主决策、民主管理、民主监督制度，促进农村基层民主健康发展。

　　第五，努力保持经济、社会、环境资源可持续发展。推进创新型宜春建设，加速科技成果转化，提高科技进步对经济发展的贡献率。巩固和提高九年义务教育，切实做好“两基”迎“国检”工作，加快发展高中教育，大力发展职业教育，支持高等院校加强内涵建设、提升办学水平，确保宜春学院通过教育部本科教学水平评估。继续实施“6666人才工程”，为全市发展提供智力支持和人才支撑。加强农村医疗卫生体系建设，帮助村卫生所、乡镇卫生院、县级综合医院改善医疗条件，提高医务人员素质；加强城市社区卫生机构服务能力建设，确保社区基本公共卫生服务覆盖95%以上的街道和居民。推进中心城区大文化产业发展，抓好文化和广电传媒体制改革，做好国家、省非物质文化遗产名录的申报工作，加快《宜春市志》修编步伐；积极开展农村和谐文化建设，倡导农村健康文明新风。广泛开展全民健身活动，提高竞技体育水平，积极承办国内外体育赛事。强化国土资源管理，实行耕地保有量、国土资源保护和管理行政首长负责制；完善土地征用、审核、批准机制，全面推行项目用地公示制，加强土地审批后续管理与督查，防止违法违规用地。强化主要污染物减排措施，严格执行环境影响评价制度；整治城区“冒黑烟”锅炉，加强火力发电、水泥、化工企业的空气污染治理，加强生活污水和工业园区污水治理，取缔饮用水源保护区内所有违法设立的排污口，实施锦江流域和樟树草溪河、袁州新坊河、南庙河水环境综合整治。强化森林资源保护和管理，禁伐天然阔叶林。加快资源节约技术的推广应用，积极开展节能降耗活动，促进循环经济发展。严格执行计划生育政策，坚决打击“两非”行为，完善利益导向机制，强化管理与服务，稳定低生育水平，提高出生人口素质。

　　第六，倾力改善民生、促进民和、确保民安。坚持以人为本，注重公平正义，从人民群众最关心、最直接、最现实的利益出发，将新增财力进一步向困难群众倾斜、向农村倾斜、向基层倾斜、向社会事业薄弱环节倾斜，全面完成民生工程建设任务，统筹解决广大群众特别是城乡困难群众的基本生活、住房、医疗、教育等保障问题，基本建立城乡义务教育免学杂费和贫困生资助体系，全面推进城乡居民基本医疗保障制度，进一步改善农村生产生活条件，不断加强社会事业的薄弱环节，尤其重视和解决群众反映比较集中的突出问题。按照省委、省政府和市委的要求，千方百计筹措资金，与中央、省财政安排的项目形成有效的资金配套；立足当前、着眼长远，注重投入的后续能力，形成长效机制；积极发挥财政资金的导向作用，引导社会资金增加民生投入。大力调整和优化财政支出结构，将更多的财政资金投向公共服务领域，在预算安排上严格按政策把资金打足，不留缺口。加强与中央、省有关部门的沟通，争取上级更多的项目、资金支持，增强财政资金的保障能力。

　　继续做好统计、档案、新闻出版工作。积极发展慈善事业和残疾人事业。切实保障妇女儿童的合法权益。认真贯彻党的民族宗教政策，充分发挥民族宗教工作在促进社会和谐中的积极作用。深入开展拥军优属活动，落实优抚政策。加强国防动员、国防教育和人民防空工作，支持驻宜解放军、武警部队和民兵预备役建设，促进双拥共建。

　　三、实现经济社会又好又快发展的主要举措

　　各位代表！实现宜春经济社会发展好中求快、快中见好，必须在全面推进中抓住关键，必须在重点突破上强化举措，必须在具体实施上务求落实。今年的政府工作，将紧扣民生为本、开放为先、创新为要、和谐为重、行政为民，致力在“五个三”上取得实质性成效。

　　第一，坚持民生为本，落实“三大保障”。大力推进就业保障。全面落实促进就业再就业的各项优惠政策，完善再就业援助制度，确保城镇新增就业人员4.52万人、净增就业人员3.6万人，新增转移农村劳动力6万人。切实加强对“零就业家庭”及“4050”人员等困难群体的就业帮扶，确保有就业愿望和能力的“零就业家庭”至少有一人就业。完善小额贷款激励机制，扩大发放规模，全年新增9500万元以上，重点推介创业项目300个。实行城乡统筹就业，建立城乡统一的劳动力资源管理制度、就业促进政策、劳动力市场、职业技能培训体系和用工管理制度。加大劳务输出力度，建立健全驻外劳务管理机构，为外出务工人员提供有效服务，确保跨省劳务输出67万人。加强职业技能培训，组织创业培训3000人，完成农村劳动力转移培训10.4万人，与园区企业对接岗位3万人。加快宜春（赣西）公共实训中心建设，确保一期工程投入使用。

　　不断改善生活保障。以非公有制企业、城镇个体工商户和灵活就业人员为重点，扩大基本养老保险覆盖面，确保参保人数达40.66万人，参保率63.1%以上，基金征缴额5.8亿元。全面实施基本养老金计发办法改革，做好企业离退休人员待遇调整，探索建立被征地农民和农民工养老保险制度。完善城乡医疗保障，积极帮助国有困难企业和集体企业职工参加医疗保险，确保城镇职工参加基本医疗保险人数达36万人，参保率53.23%。推进城乡合作医疗，在新型农村合作医疗市域全覆盖的基础上，确保农民参合率85%以上；采取财政补助的办法，建立城镇居民合作医疗保险制度，确保参合人数达到24万人，参合率33%以上。完善城乡社会救助体系，切实做好城乡低保工作。加强城乡大病医疗救助，对患有规定救助病种的7.4万名农村困难群众和2.03万名城市困难群众实行救助。继续实施“蓝天计划”，进一步改善孤残儿童的生存生活条件。加快农村敬老院、社会福利院和光荣院建设，农村“五保”对象集中供养率达到80%以上。启动中心城区老年公寓建设，力争年内建设市社会福利中心。大力推进经济适用房建设，确保中心城区建设8万平方米、受益家庭1000户，9个县市建设10万平方米、受益家庭1250户；进一步推进　廉租房制度，以实房租住或货币补贴形式，帮助更多城市特困群众解决住房困难，确保中心城区受益家庭826户，9个县市受益家庭496户。

　　积极健全教育保障。全面实行城乡义务教育免学杂费和贫困生免教科书费，贫困寄宿生生活费补助标准提高到300元。提高义务教育阶段公办中小学公用经费标准，农村小学年生均经费提高到74.5元，城市小学提高到80元；农村初中年生均经费提高到94.5元，城市初中提高到110元。建立非义务教育阶段特殊困难家庭学生资助政策体系，对公办高中的特殊困难家庭学生，每人每年补助800元；对职业高中、普通中专、成人中专、技工学校的特殊困难家庭学生和残疾学生，每人每年补助1000元；对本市考取全日制普通高等学校（不含独立学院和民办高校）的特殊困难家庭学生，每人一次性补助5000元。认真实施农村中小学校舍维修改造和基础设施改造；两年内新增宜春学院、宜春职业技术学院大专以上在校生生均经费400元。将农民工子女义务教育纳入流入地政府教育发展规划，以全日制公办中小学校接收为主，享受与城镇居民子女同等待遇。

　　第二，坚持开放为先，推进“三大战役”。加速开发区的扩张，打好工业强攻战。扩大区域面积，将宜春经济开发区规划面积拓展至150平方公里，管辖面积扩大到84平方公里；理顺管理体制，授予管委会相应的审核审批权、行政管理权；建立健全财政体制和投入机制，将市中小企业信用担保中心划归开发区管理，每年由市财政安排适量资金充实担保基金，市工业和民营经济发展专项资金主要用于开发区企业发展和招商安商奖励；赋予“特区”地位，制定职责明确、办事高效、服务优质的规章制度，建立部门负责人和工作人员专职服务机制，营造最适合企业生存与发展的“生态”环境。今年新引进固定资产投资亿元以上项目5个，新增投产企业20家。各县市区主动对接“心圈廊”战略及“强攻战”部署，加强工业园区建设，完善基础设施和生活服务设施，健全服务机制，促进产业聚集和企业集群，提高土地利用强度、单位土地面积投资强度和投入产出率。力争园区新增固定资产投资500万元以上的投产企业140家，实现主营业务收入348亿元，上缴税金20亿元。

　　加速新城区的启动，打好城市扩张战。按照“高起点规划、高标准建设、高水平管理、高效益经营”的原则，修编未来五年宜春中心城土地利用和建设规划，完成宜阳新区30平方公里的控制性详细规划和4平方公里核心区的修建性详细规划，科学构建城市发展框架。坚持规划就是城市建设的“宪法”，强化实施规划的法律监督和效能监察，严格执行城市规划管理“十不准”，严肃查处违规违法建设，维护规划的权威性。加快行政中心主体工程建设，不断完善宜阳新区路网及配套工程，有效提升城市承载功能。合理实施“北拓南控、南改北建”策略，在集中力量建设新城区的同时，有序推动老城区改造。加快中心城污水处理厂、环卫基础设施、建筑垃圾消纳处置场建设，继续实施防洪工程和城南排水工程；认真抓好中心城区第五期80条小街小巷改造，城区内所有公厕免费开放，供水一户一表改造和供水管网向城郊覆盖，完成燃气改气改制工作，进一步完善公交网络。以创新中国成立家卫生城市为主线，强化城市管理，完善综合执法，创新环卫管理体制，促进文明社区创建，做好第二届省级文明城市申报工作。抓紧县市土地利用和城市总体规划的编制，推进以宜春中心城区为中心、以县市城区为次中心、以乡村集镇为节点的城镇群建设。继续加强县市创建省级以上园林城、卫生城等工作。

　　加速风景区的创建，打好旅游升温战。加快明月山温泉风景名胜区的开发、建设与营销，积极创建生态优良、服务配套、管理规范的国家AAAA级景区，打响“奇山、妙泉、名禅、神农”品牌。着力建好月亮湖和月亮湾文化乐园，积极引进国际禅修中心、高品质矿泉水厂、里布水库建设项目，完成游客服务中心、仰山栖隐禅寺主体工程建设和温潭公路拓宽改线，开工兴建天工开物园、温泉古井公园、温泉养生文化公寓和万吨地热水供应系统，整治美化温汤河，开通中心城区至温汤、明月山公交线路。充分发挥明月山温泉风景名胜区建设的带动作用，加快“中国优秀旅游城市”创建步伐，整合全市旅游资源，捆绑成势，连点成线，着力打造绿色山水、禅宗文化、生态农业和农耕文化、红色文化四条精品旅游线路。加强县市旅游基础设施建设，完善旅游服务功能，促进旅游产业快速发展。力争接待海外游客1.3万人次，实现旅游外汇收入500万美元以上；接待国内旅游者460万人次，实现国内旅游收入26亿元以上。

　　第三，坚持创新为要，深化“三项改革”。推进农村综合改革。稳步推进乡镇机构改革，进一步转变乡镇政府职能，加强编制实名制管理；建立和完善政府投入办学、各级责任明确、财政分级负担、经费稳定增长的农村义务教育经费保障机制，合理配置教育资源；加快推行“乡财县代管”，加强乡镇财政管理，积极化解乡村债务，防止产生新的债务，严禁向村一级下达税收任务。规范农村土地流转和“一事一议”制度，确保农民负担不反弹。完善农村信用社产权制度改革，提高农村金融服务水平。继续抓好林业产权制度改革扫尾工作，加快基层林业“三防”体系建设。推进农村小型水利工程管理体制改革，全面完成农村用水协会组建任务。

　　深化企事业单位改革。按照市直事业单位改革实施意见的总体要求，认真做好事业单位的“三定”方案编制和改革试点工作，积极推行事业单位全员聘用制。健全事业单位社会保障体系，在继续实施自收自支事业单位养老保险统筹的基础上，逐步将差额拨款事业单位纳入养老保险体系。各县市区要按照上级有关要求，争取在事业单位改革上取得新突破。全面实施政府收支分类改革，深化部门预算、国库集中收付制度改革，积极稳妥地实施工资制度改革，严格实行政府采购“管采分离”，落实预算外资金“收支两条线”规定。健全国有资产监管体系，确保国有资产保值增值。深化粮食企业改革，不断完善“国有控股、调控有力、自主经营、效益良好”的粮食企业体系。继续做好国企改革工作，抓紧启动长青厂和汽运公司改制，积极推进三九生化战略重组，力争江特公司上半年上市。

　　加快市区管理体制改革。按照增强合力、提高效率和充分调动两级积极性的原则，合理调整市、区两级事权和财权，促进两级财政的共同发展。巩固和完善“两级政府、三级管理、四级网络”的“联创联建、综合执法、社区自治”管理模式，健全城市管理长效机制。根据依法与效能一致的原则，以及新形势、新任务的要求，对公安、国土、教育、卫生等行政体制进行优化，带动和支持袁州区参与新城区建设，在规划上协调，在政策上扶持，在进程上同步。

　　第四，坚持和谐为重，夯实“三大基础”。全力抓好安全稳定。认真贯彻“安全第一、预防为主、综合治理”的方针，强化安全生产工作措施，始终保持安全生产高压态势。进一步落实安全生产责任制，强化企业安全生产自我约束和主体责任，加大安全生产投入，完善安全生产设施，加快安全生产的规范化、制度化建设。深化煤矿、花炮、道路交通、消防、食品等安全专项整治，强化监管，严格执法，严肃查处违法违纪和失职渎职行为，切实消除各种安全隐患，坚决遏制重大事故的发生，确保安全生产形势平稳。高度重视高等院校和民办学校的思想政治工作，进一步强化内部管理，构建和谐平安校园。建立健全社会预警体系和应急救援、社会动员机制，提高多发性事件的稳控能力和突发性事件的处置能力。加强自然灾害监测预报和防治救助体系建设，提高防灾、减灾、抗灾、救灾能力。

　　深入开展社会治安综合治理。按照“属地管理、分级负责，谁主管、谁负责”的原则，健全综治领导责任机制，加大责任追究力度。积极推进平安创建活动，全市80%的县市区、90%以上的乡镇村达到和谐平安建设标准。健全矛盾纠纷排查调处机制，完善基层矛盾纠纷调处中心、乡镇（街道）和谐平安联动中心，最大限度地把矛盾解决在基层、化解在萌芽状态。深入开展“严打”斗争，严厉打击各种刑事犯罪和经济犯罪，着力整治突出治安问题。健全群防群治机制，推进社会治安防控体系建设，增强全民法制观念和守法意识，努力为人民群众创造安全、安定、祥和、有序的工作生活环境。

　　高度重视人民群众来信来访工作。健全利益协调和权益保障机制，想问题、办事情从群众利益出发，落实涉及群众利益的各项政策，从源头上减少信访问题的发生。不断完善信访工作机制和群众诉求表达机制，畅通信访渠道，完善市长专线及网络建设，及时为群众提供信访服务。创新息访方法和手段，针对群众不同的诉求，按照“合法合规、依情依理”的原则，妥善处理各类信访问题。坚持实行信访重点地区管理制度，认真开展重复信访问题专项治理，有效遏制越级访、重复访和非正常访。深入贯彻《信访条例》，双向规范信访、接访行为，维护正常的信访秩序。进一步落实信访工作领导责任制，完善各级领导信访接待日、领导包案处理重点信访问题等制度，加大督办力度，切实维护群众的合法权益。

　　第五，坚持行政为民，提升“三种能力”。提升贯彻决策的执行力。不断强化各级政府和工作人员的大局观念。提高对上级方针、政策、部署的理解力和执行力，确保政令畅通。切实加强自我约束，自觉接受人大及其常委会的法律监督、政协的民主监督和社会舆论监督，保障执行结果符合人民意愿、体现决策意图。积极推进政府决策的法制化、科学化、民主化，使决策更符合经济社会发展实际，更贴近基层群众需求。不断提升各级政府领导和工作人员的自身素质。增强忧患意识，始终保持开拓进取的锐气；增强公仆意识，始终牢记全心全意为人民服务的宗旨；增强节俭意识，始终发扬艰苦奋斗的精神。进一步提升驾驭市场经济、破解发展难题的能力，提升协调各方关系、化解各种矛盾的能力，提升促进自主创新、领导科学发展的能力，提升主持公平正义、依法行政办事的能力。坚持反腐倡廉、从严治政，加强审计监督和行政效能监察，自觉践行八种良好风气，进一步规范行政权力运行，严肃查处损害群众利益和败坏政府形象的行为，切实把政风建设和廉政建设责任制落到实处。

　　激发开拓局面的创造力。主动顺应时代潮流，积极应对挑战考验，不断提高创新能力。创新发展意识，把坚持科学发展、创建和谐社会的思想，创造性地贯穿到政府工作中，力求使各项工作符合科学发展观与构建和谐社会的要求，加快转变经济增长方式、改革体制机制、优化干事创业环境。创新工作方法，克服“等、靠、要”的惯性思维和传统做法，注重用新理论、新思想、新知识充实自己，善于用经济手段和市场手段开展工作，破除“条条框框”，减少繁文缛节，把精力真正用在抓改革、促发展、保稳定的大事上，用在解决人民群众生产生活的难事上。创新工作举措，注重研究政策与举措，做到有的放矢；善于取长补短，学习借鉴外地的先进经验，在工作中大胆探索，努力走出新路子、取得新成效。

　　　强化真抓实干的操作力。面对伟大的事业和艰巨的重任，各级政府工作人员不仅要有不当“旁观者”的品格，更要有善做“战斗员”的才能，从每件事情做起，从每个人做起，把有效的操作力贯穿于实施、落实、见效的全过程。在贯彻和执行上级决策上，善于“结合”、“转化”、“到位”，而不生搬照套。在实施具体任务中，既重深思熟虑，注意工作的上下衔接、左右兼顾；又重争分夺秒，树立强烈的时间观、效率观、质量观。在处理和解决实际问题时，善于“钻进去”攻坚克难，加强调查研究，领会上情、了解外情、吃透下情，化解工作中的阻力与障碍，不达目的不罢休；乐于“沉下去”真抓实干，深入到经济建设前沿，掌握第一手资料，扑下身子抓落实，全力以赴促发展；勇于“豁出去”承担责任，恪尽职守，亲力而为，决不因上交矛盾、下推问题而延误决战时机和发展大计，以科学的态度和求实的精神做好各项工作。

　　各位代表！宏伟蓝图令人鼓舞，光荣使命催人奋进。让我们紧密团结在以胡锦涛同志为总书记的党中央周围，在省委、省政府和市委的坚强领导下，进一步凝聚全市人民的智慧和力量，拼搏进取，奋发图强，在新的起点上再创新的辉煌，以优异成绩向党的十七大献礼！
